# Influences on the duration and success of out-of-hospital resuscitation of geriatric patients over 80 years of age - a retrospective evaluation

**DOI:** 10.1186/s12873-024-01099-3

**Published:** 2024-10-10

**Authors:** Nils Heuser, Dennis Rupp, Susanne Glass, Martin Christian Sassen, Astrid Morin, Christian Volberg

**Affiliations:** 1 Department of Anesthesiology & Intensive Care Medicine, Faculty of Medicine, Philipps University of Marburg, 35043 Marburg, Germany; 2 EMS Mittelhessen, German Red Cross, Marburg, Germany; 3 Department of Emergency Medicine, DGD Diakonie-Hospital Wehrda, Hebronberg 5, 35041 Marburg, Germany; 4 Department of Hazard Prevention, District of Marburg-Biedenkopf, Im Lichtenholz 60, 35043 Marburg, Germany; 5 Research Group Medical Ethics, Faculty of Medicine, Philipps University of Marburg, 35043 Baldingerstraße, Marburg, Germany

**Keywords:** Out of hospital cardiac arrest, Cardiopulmonary resuscitation, Elderly, Termination of resuscitation, Geriatrics

## Abstract

**Background:**

Society is experiencing an increasing shift in the age distribution and accordingly, increased resuscitation rates of patients over 80 years and older. In 2022, more than 34% of people resuscitated in Germany were older than 80 years, although older age is considered a poor predictor for the outcome of cardiopulmonary resuscitation (CPR). Professional societies provide ethical recommendations on when resuscitation may be considered futile and should be terminated. However, the extent to which these recommendations are implemented is unclear.

**Methods:**

Retrospective evaluation of pre-hospital documentation of out-of-hospital resuscitations in patients ≥ 80 years of age in the period 01/01/2014–12/31/2022 in one German county combined with data of the German Resuscitation Registry. For statistical testing, the significance level was set at *p* < 0.05.

**Results:**

In total 578 cases were analyzed. Return of spontaneous circulation (ROSC): 26% (*n* = 148). Survival to discharge: 6.1% (*n* = 35). Median CPR duration: 17 min (10–28 min). The older the patients were, the worse the survival rate (*p* = 0.05) and the shorter the time to termination (*p* < 0.0001). No patient over 90 years of age was discharged alive. Resuscitation was also significantly shorter until termination with poorer ASA (American Society of Anesthesiologists) score (*p* < 0.001). Residents resuscitated significantly longer than specialists (*p* = 0.02). In surviving patients, there was a significant correlation between short CPR duration and good cerebral performance category (CPC) value: Median CPC1/2 = 5 min [3–10 min] vs. CPC 3/4 = 18 min [10–21 min]; *p* = 0.01.

**Interpretation:**

Old age and poor health status is associated with shorter CPR duration until termination and older age is associated with poorer prognosis in out-of-hospital cardiac arrest (OHCA) concerning the possibility of return of spontaneous circulation (ROSC) and survival. A short resuscitation time is associated with a better CPC value. Therefore, when resuscitating patients over 80 years of age, even greater care should be taken to ensure that reversible causes are quickly corrected in order to achieve a ROSC and a good neurological outcome. Alternatively, resuscitation should be terminated promptly, as good survival can no longer be guaranteed. Resuscitation lasting more than 20 min should be avoided in any case, in line with the termination of resuscitation (ToR) criteria.

## Background

Out-of-hospital cardiac arrest (OHCA) is a highly researched topic in emergency medicine. The incidence of pre-hospital resuscitations in Germany in year 2022 was 57.3 per 100.000 inhabitants, which extrapolates to around 60,000 OHCAs per year [1]. More than one third of these patients were over 80 years of age. This means an increase of 6.6% compared to 2014 [1]. In addition, the proportion of the population aged over 60 in Germany is predicted to rise up to 35% by 2030 and the number of people aged 80 or over is about to rise from 4.4 million in 2013 to 10 million in 2050 [2, 3]. This reflects the importance of research on pre-hospital resuscitation of old patients. More age-related illnesses and a “geriatrization” of the patient clientele show that the proportion of old patients is increasing [2, 4, 5]. As age is regarded a predictor of a futile outcome of cardiac arrests in and out of the hospital [6–8], these numbers are also effecting the setting of prehospital cardiopulmonary resuscitation (CPR). In addition to age, there are many other factors that influence out-of-hospital CPR. Professional societies such as the American Heart Association (AHA) or the European Resuscitation Council (ERC) cite further predictors that suggest a futile outcome of resuscitation measures and which, from an ethical and moral point of view, can help to answer the question of whether resuscitation should be discontinued or not initiated in the first place [9, 10]. In this context, so-called “termination of resuscitation” (ToR) rules have been developed, which can help to decide when out-of-hospital resuscitation can be terminated [6, 11]. In our study, we focused exclusively on patients aged ≥ 80 years and the influence of various factors (age, duration of resuscitation, initial rhythm, pre-emergency-status, specialty/qualification of the emergency physician on the course and outcome of resuscitation in these patients). For one thing we observed if increasing age affected the probability of achieving return of spontaneous circulation (ROSC) and, furthermore, the probability of surviving with a good neurological outcome. Apart from that, it is of interest to what extent the age and pre-existing condition of the patient influences CPR duration until the emergency physician on scene decides to end the resuscitation and if there are factors concerning the treating emergency physician, that have an influence on that decision.

## Methods

After the positive vote of the local ethics board from 03/14/2023 (file number: 23–98 RS; ethics board of the Philipps University of Marburg, Germany), records of all OHCA events that occurred in a German county with about 250,000 inhabitants between 1 January 2014 to 31 December 2022 were analysed retrospectively. Cases were excluded from analysis if irreversible death had already occurred, and CPR had not been initiated.

### Data collection

Analysed data are collected routinely for structured CPR feedback as part of the ambulance services’ quality management (German Red Cross, EMS Mittelhessen, Germany). The data is obtained anonymously. Patient names or other personal information (e.g. address) are not stored. Data contain clear summaries of every CPR in the county, taken from electronic report and defibrillator recordings, manually investigated by only one paramedic. Results are compared to guideline specifications and made available to every involved EMS (Emergency Medical Service) team member for continuous improvement of CPR quality. Data is supplemented by entries in the German Resuscitation Registry (GRR) in order to collect outcome parameters.

### Statistical analysis

A good neurological outcome was defined as a Cerebral Performance Category (CPC) score of 1 (good cerebral performance (normal life)) or 2 (moderate neurological disability (disabled but independent)). Scores of ≥ 3 (severe cerebral disability/coma/vegetative state/brain death) were defined as a bad neurological outcome [12]. The ASA (American Society of Anesthesiologists) score was used to estimate the health status of the patients prior to the event. Considering the main objectives, various subgroups were formed and correlated with dependent variables. The main subgroups were the different age classes, but also a categorization into ROSC/ToR, different ASA scores, different neurological outcomes and factors concerning the emergency physician on scene was made. Variables were the duration of resuscitation, the percentage of ROSC and the numbers of survival to discharge. Statistical analysis was performed using Microsoft Excel^®^, version 16.80 (Microsoft, Redmond, VA, USA). Statistical calculations were performed for those patients with documented CPR duration. The mean, standard deviation, minimum and maximum as well as the corresponding confidence intervals were calculated as descriptive measures. For non-normally distributed variables, the corresponding non-parametric measures of position were calculated (median, interquartile range). Any differences were tested using a t-test, analysis of variance (ANOVA) and chi-square test with a significance level of *p* < 0.05. When performing pairwise comparisons Holm-Bonferroni correction was incorporated, resulting in adjusted *p*-values to reduce the probability of Type I errors. For these comparisons, values of *p* considered to be statistically significant are separately indicated at the corresponding location.

## Results

From 01/01/2014 to 12/31/2022, on a total of 578 patients ≥ 80 years resuscitation was attempted. Average age was 85 ± 3.97 years. Patients aged 80–84 years: *n* = 291 (50.3%); 85–89 years: *n* = 204 (35.3%); ≥ 90 years: *n* = 84 (14.4%).

Not all data were available for all patients. CPR duration was available in *n* = 546 (94.5%) cases, qualification of the treating emergency physician in *n* = 541 (93.6%) and specialty of the treating emergency physician in *n* = 540 (93.4%) cases. Pre-emergency ASA score was documented for *n* = 522 (90.3%) patients. The initial rhythm was documented for *n* = 577 (99.8%) patients. For the remaining subgroups, data were available from all 578 patients. The comprehensive demographic data of the patients are shown in Table 1.

**Table 1:** Demographic data of the included patients (age, gender, CPR-Duration, ASA score, percentage of ROSC/ToR/Survival to discharge and the CPC score on discharge have been observed)

	80–84 years	85–89 years	≥ 90 years
n (total: 578)	291 (50.3%)	204 (35.3%)	83 (14.4%)
Gender			
male [%]	61.0 (*n* = 177)	54.4 (*n* = 111)	39.8 (*n* = 33)
female [%]	39.0 (*n* = 113)	45.6 (*n* = 93)	60.2 (*n* = 50)
Median CPR Duration overall [min.]	18 [10–29.75]	18 [10–28]	15 [7–21]
Initial Rhythm			
Shockable [%]	14.8 (*n* = 43)	16.2 (*n* = 33)	4.8 (*n* = 4)
Non-Shockable [%]	85.2 (*n* = 248)	83.8 (*n* = 177)	94.0 (*n* = 78)
Pre emergency health status			
ASA 1 [%]	2.1 (*n* = 6)	1.5 (*n* = 3)	0 (*n* = 0)
ASA 2 [%]	18.6 (*n* = 54)	17.6 (*n* = 36)	16.9 (*n* = 14)
ASA 3 [%]	62.9 (*n* = 183)	56.9 (*n* = 116)	61.4 (*n* = 51)
ASA 4 [%]	9.3 (*n* = 27)	11.3 (*n* = 23)	10.8 (*n* = 9)
Outcome			
Any ROSC [%]	42.6 (*n* = 140)	31.9 (*n* = 65)	28.9 (*n* = 24)
ROSC on Arrival [%]	29.9 (*n* = 87)	20.6 (*n* = 42)	22.9 (*n* = 19)
Prehospital ToR [%]	70.1 (*n* = 204)	79.4 (*n* = 162)	77.1 (*n* = 64)
Survival to Discharge [%]	9.6 (*n* = 28)	3.4 (*n* = 7)	0 (*n* = 0)
CPC 1/2	6.2 (*n* = 18)	3.4 (*n* = 7)	0 (*n* = 0)
CPC 3/4	2.7 (*n* = 8)	0 (*n* = 0)	0 (*n* = 0)

**Fig. 1:**
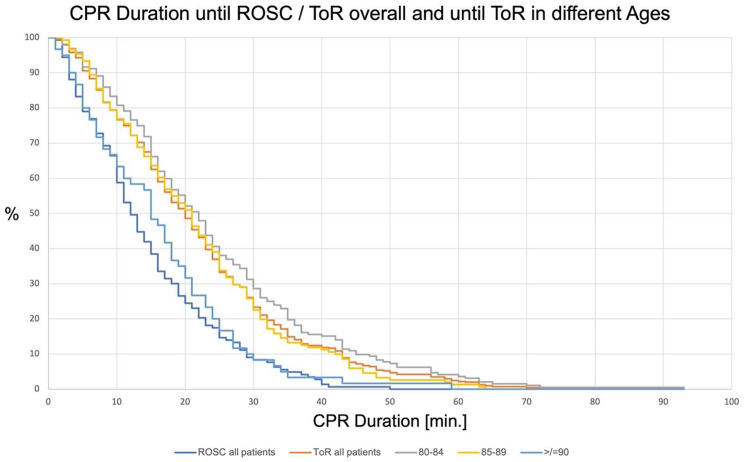
Overall CPR duration until ROSC/ToR; *p* < 0.001 and CPR duration until ToR in different age groups (*p* < 0.001)

Concerning the chances of survival, we observed that 35 patients (6.1%) survived to hospital discharge of which *n* = 33 (5.7%) had been admitted to hospital with ROSC while *n* = 2 (0.3%) had ongoing resuscitation on admission. 28 patients (80.0%) with documented CPC score were younger than 85 years (81.8 ± 1.4 years), seven patients (20.0%) were older than 85 years (86.7 ± 1.7 years). Two patients (0.3%) had no documented CPC score. No patient over 90 years was discharged from hospital alive. The influence of the CPR duration on the chances of survival are presented in Table 2 / Fig. 1.

**Table 2:** Observed Parameters / Statistical Data

	80–84 years		85–89 years			≥ 90 years		*p*-value
n	274 [47.4%]		193 [35.3%]			79 [14.5%]		
Median CPR Duration overall [min.]	18 [10–29.75]		18 [10–28]			15 [7–21]		0.02
Median CPR Duration until ToR [min.]	22 [13.75–32.25]		21 [12–30]			15 [7–23]		< 0.001
ROSC on Arrival [%]	29.9 [*n* = 87]		20.6 [*n* = 46]			22.9 [*n* = 19]		0.05
Survival to discharge [n]	28		7			0		< 0.001
	**ROSC**			**ToR**				***p*** **-value**
Median CPR Duration until ROSC/ToR [min.]	12 [7–20]			20 [11.5–30]				< 0.001
	**CPC 1/2**		**CPC3/4**		**Intrahospital Death**			***p*** **-value**
n	23 [4%]		7 [1.2%]		113 [19.6%]			
Median CPR Duration [min.]	5 [3–10]		18 [11–21]		14 [9–22]			0.001 / <0.001
Age (a)	82 ± 2.8		82.2 ± 1.1		85 ± 4			0.19
	**Resident**			**Specialist**				***p*** **-value**
n	190 [35.1%]			351 [64.9%]				
Median CPR Duration overall [min.]	20 [11–30]			16 [9–26]				0.02
Median CPR Duration until ToR [min.]	22 [10–32]			18.5 [11–29]				0.05
	**Anesthesia**	**Internal Medicine**		**Surgery**			**Other (e.g. General Practitioner)**	***p*** **-value**
n	233 [43.1%]	153 [29.3%]		62 [11.5%]			87 [16.1%]	
Median CPR Duration overall [min.}	19 [12–29]	16 [9.25–25]		21 [7–30.75]			16.5 [9–25,5]	0.09
Median CPR-Duration until ToR [min.]	21 [2–30]	28 [10–30]		25 [9–35]			17 [10–26]	0.09
	**ASA I**	**ASA II**		**ASA III**			**ASA IV**	***p*** **-value**
Median CPR Duration overall [min.]	19 [15–25]	23 [12–32]		17 [10–27]			14 [7–22]	0.03
Median CPR Duration until ToR [min.]	23 [17–27.5]	26.5 [17–37]		20 [12–30]			14 [7.25–20]	< 0.001
Percentage of ROSC [%]	22.2 [*n* = 2]	34.6 [*n* = 36]		26.3 [*n* = 92]			18.6 [*n* = 11]	0.15

The relation between different factors (age, neurological outcome, qualification / specialtity of the treating emergency physician, ASA-Score) and the median CPR duration overall and until ToR were observed. The median CPR-duration until ROSC was compared to the median CPR-duration until ToR and also the percentage of ROSC in different age groups and of patients with different ASA-Scores, as well as the ages of patients with different neurological outcomes were evaluated

86.1% of the patients initially had a non-shockable rhythm (53.2% asystole, 32.9% PEA) and 13.9% had a shockable rhythm (13.0% VF, 0.9% PVT). Of the surviving patients, 40% had a shockable rhythm (VF) and 60% a non-shockable rhythm (22.9% asystole, 37.1% PEA).

**Fig. 2:**
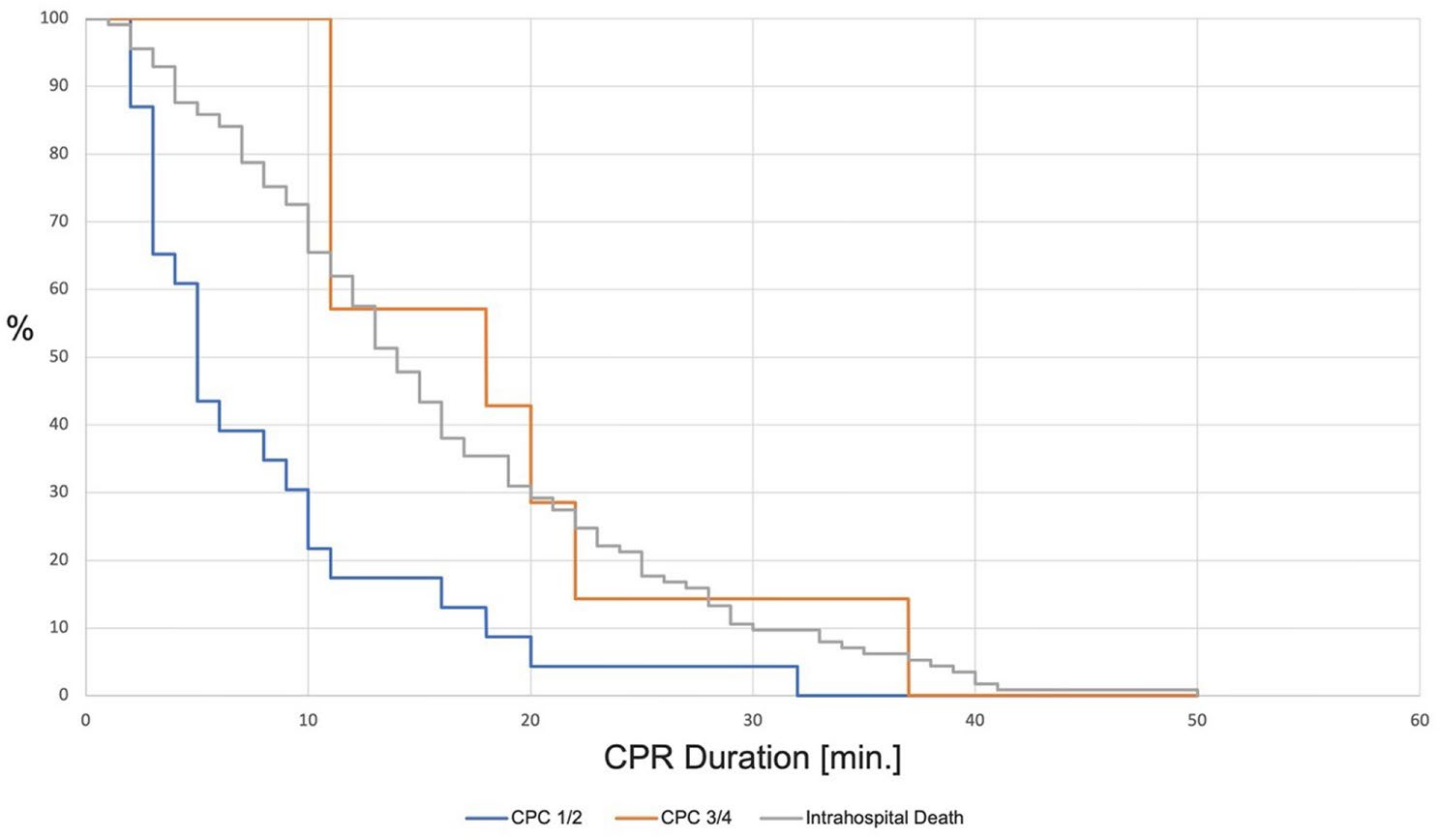
Influence of duration of resuscitation on neurological outcome. Duration: CPC 1/2 vs. CPC 3/4; *p* = 0.01. Duration: CPC 1/2 vs. death in hospital; *p* < 0.001

**Fig. 3:**
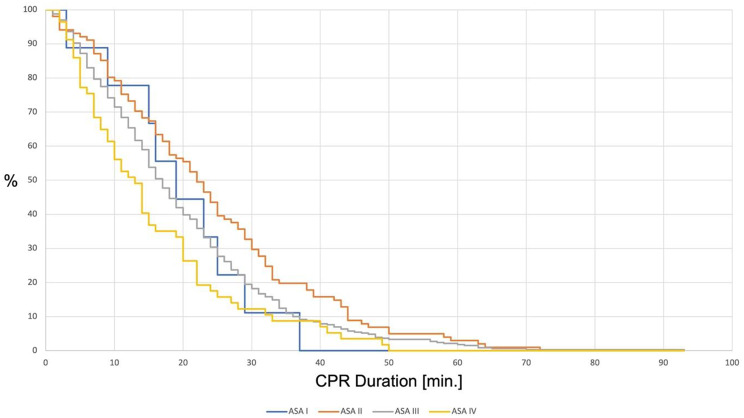
CPR duration of patients with different ASA scores until termination of resuscitation (*p* < 0.001) and health status of the patient collective before OHCA. 70% of the patients had an ASA score of ≥ 3 prior the event

Regarding the factor CPR duration, we observed that the median duration of resuscitation was 17 min [10–28 min]. A detailed view of the CPR duration until ROSC or ToR in different age groups is shown in Table 2; Fig. 1. The influence of CPR duration on the neurological outcome is shown in Fig. 2. We also observed the influences of the speciality and qualification of the emergency physician and the documented ASA score of the patients on the CPR duration. 35.1% (*n* = 190) of the treating emergency physicians were residents, 64.9% (*n* = 351) were specialists. The detailed data showing the qualification and speciality in relation to the CPR duration overall and until ToR are shown in Table 2. Of a total of 496 patients (85.8%), both ASA score and CPR duration were documented. On average, these patients were 85 ± 4 years old. In most patients, an ASA score of III was documented (66.3%, see Fig. 3). Data showing the relation of patients with different ASA scores to the median CPR duration and the percentage of ROSC are shown in Table 2; Fig. 3.

## Discussion

Many studies to date have investigated age, frailty, the duration of resuscitation and many other factors and their influence on the outcome of resuscitations in and out of the hospital. However, these factors have often been studied independently of each other, in relation to a very heterogeneous patient population or exclusively in relation to in-hospital resuscitations [7, 12–16]. In our study, we focused exclusively on patients over 80 years of age and analyzed contextual factors that influenced prehospital resuscitations and the outcome of these patients. The topic of out-of-hospital resuscitation of very old people is extremely complex in emergency medical practice. This concerns medical as well as ethical and moral aspects. Medically, as patients in old age often have multiple and chronic pre-existing conditions, ethically and morally, as the question arises as to whether death in this age group is merely the natural end of life and to what extent these people are actually helped during resuscitation or whether their suffering is prolonged and natural death is artificially delayed in case of doubt [2].

Our analysis, as well as former observations, shows that age is a good predictor for a futile outcome of the resuscitation, concerning both, achieving ROSC and surviving until hospital discharge [6–8]. Yet, unlike other studies, we did not observe that the probability of achieving a good neurological outcome (CPC 1 or 2) decreases with increasing age, which could be related to the fact that only very few patients survived at all [7, 17, 18]. No patient older than 90 years of age survived until discharge of the hospital and most of the patients who survived were younger than 85 years of age (28 out of 35 surviving patients). Concerning CPR duration, patients who were resuscitated for a shorter time were significantly more likely to achieve ROSC than those with longer CPR duration, as shown in former studies [19]. We can also demonstrate a correlation between short resuscitation times and the likelihood of a good neurological outcome. The patients with a good neurological outcome (CPC 1 or 2) were resuscitated significantly shorter than those with a CPC of 3 or 4 (*p* = 0.01) or those who did not survive (*p* < 0.01). This is analogous to former observations, although there are also other studies showing that a better neurological outcome can be achieved with longer resuscitation (30–40 min). However, these studies did not only refer to patients of older age [13–15, 19–22]. We also showed that patients were resuscitated significantly shorter with increasing age. This was also evident in the subgroup of patients in whom resuscitation was terminated. Patients were also resuscitated significantly shorter, or resuscitation was terminated significantly earlier the worse their ASA score was. This is in line with previous studies which showed that CPR duration decreases with increasing frailty and a poorer health status [23, 24]. Further studies showed, that these are also predictors of a futile OHCA outcome, which we could not observe probably due to the small number of surviving patients [16, 25, 26].

In our study, 86.1% of the patients had a non-shockable initial rhythm (asystole, pulseless electrical activity), which is a lot more compared to all out-of-hospital resuscitations in Germany in 2022 (79,4%) [1]. Only 13.9% had a shockable rhythm (ventricular fibrillation, pulseless ventricular tachycardia). Of the surviving patients, however, 40% initially had a shockable rhythm.

This, as well as the negative influence of a longer CPR duration on the outcome, shows the relevance of quickly ruling out and treating reversible causes in out-of-hospital cardiopulmonary resuscitation of patients over 80 years of age and patients with a poor health status and then, in the absence of successful therapy, also quickly deciding in favor of terminating the resuscitation. According to our evaluation, the longer the resuscitation period, the lower the probability of survival with a good neurological outcome. Considering that prolonged resuscitation in patients aged 80 or older are only expected to have a small chance of survival, the question arises as to whether, at a certain age, the death of a very old person should be regarded as the natural end of life. If one is aware of the ethical principles of medical work (autonomy, beneficence, non-maleficence, justice) and then considers that the probability of survival with a favourable outcome is very low, the question arises as to whether a low chance of survival does not violate the ethical principle of non-maleficence, according to which hopeless measures should not be carried out in order to protect the patient [27–30].

Regarding emergency physician factors, we can show in our cohort, that specialist physicians resuscitate for a significantly shorter time than residents, which leads to the conclusion that they were quicker to decide that resuscitation was hopeless and to discontinue it. This is in line with previous studies, which showed that the higher the level of training, the easier it was for physicians to make end-of-life decisions [31]. In German hospitals it is common practice that junior physicians are not allowed to make such decisions “independently” within the hospital and must always consult a specialist. Pre-hospital, however, it is required that the emergency physician (whether resident or specialist) must decide to terminate or continue CPR. This is particularly critical in view of the fact that in emergency medicine critical decisions have to be made under enormous stress and in a short time and emergency physicians are under high pressure to act [32]. In view of the ever-increasing number of geriatric patients, one could conclude that it would be useful to provide emergency physicians with tools, such as an algorithm for terminating resuscitation like the ToR criteria and AHA recommendations, to facilitate such decisions. The updated 2022 ToR criteria include initial asystole, unobserved circulatory arrest, CPR duration of > 20 min and failure to achieve prehospital ROSC. These show a predictive power of > 99% for 1-month mortality [11]. However, we conclude that these tools should also consider age, health status and other factors that prove to be good predictors for a futile outcome of resuscitation in patients of older age. It is important to note that individual factors, such as age, should not be an exclusive criterion as it would be discriminatory. Previous studies have shown that the accuracy of mortality prediction increases when several factors are present, such as the initial heart rhythm, which has an even worse predictive value than age [11, 20, 33, 34]. This is also reflected in our study, where we observed a higher rate of non-shockable rhythms in patients over 80 years of age than in general in Germany, which is already a good predictor for a poor outcome of resuscitations. In addition, the surviving patients were more likely to have a shockable rhythm. However, it is not clear to what extent such or similar recommendations for action are already used in everyday practice in the management of out-of-hospital cardiac arrests.

The training of emergency physicians should also focus on the treatment of geriatric patients, as this patient group accounts for an increasing proportion of emergency medical interventions and such patients are more frequently confronted with “end-of-life” decisions [2–5].

## Limitations

A limitation of this study is the retrospective design of the study from a single ambulance service area in Germany. The study included 578 patients who were resuscitated by ambulance staff and emergency physicians in one German county. It remains to be seen whether the results can be transferred to other rescue services without further ado. The emergency physician system in this EMS is mostly based on physicians from local hospitals (university hospitals and in some cases standard care providers). It is conceivable that other results could also be measured in a differently structured emergency physician system. The same applies to non-emergency physician-based systems, such as those in Anglo-American countries. With regard to the data collected, it must be mentioned that not all data were available from all 578 patients who were examined in the various subgroups. As a result, the patient collective varies between *n* = 496 and *n* = 578 patients in the different subgroups. A further limitation is the low number of observations in some of the subgroups (e.g. survivors (*n* = 35)), which made it difficult to carry out a meaningful analysis. This study analyzed the resuscitation records of the emergency medical services and the treating emergency physicians as well as entries in the German Resuscitation Registry. We must assume that these entries were always made as conscientiously as possible. Possible errors in the entries cannot be ruled out in retrospect. The underlying event that led to the cardiac arrest was not considered in our data collection, as the exact genesis often cannot be diagnosed in the prehospital setting and the data would therefore be too speculative. The neurological outcome of the patients was measured based on the CPC status of the surviving patients on discharge from hospital. Due to their age, it is conceivable that the patients already had a reduced CPC status before the event. This could not be determined on the basis of the available data. No information is available on longer-term survival, the course of neurological status or the patients’ quality of life.

## Conclusion

In this study, the factors age, ASA score, level of training and specialty of the emergency physician and their influence on the duration of resuscitations, their outcome (ROSC/ToR) and the neurological outcome of the patients were investigated. Other factors were not taken into account. It must be said that age and health status of patients prior to cardiac arrest are only two of many factors that influence resuscitations and their outcome. Nevertheless, in our study, older age showed to be a good predictor of a poor outcome of prehospital resuscitations. This applied to the probability of achieving ROSC and to survive with a favorable neurological outcome. With regard to CPR duration, we were able to show that, on elderly patients older than 80 years of age, shorter resuscitations are associated with a higher probability of survival and that prolonged resuscitation attempts are more likely to end fatally. Patients with shorter durations of CPR prior to ROSC were more likely to survive with good neurological outcomes at discharge. If ROSC is not achieved within this timeframe consideration should be given to cessation of further resuscitation attempts. Patients with a bad ASA score prior to the event were resuscitated shorter until termination of resuscitation was initiated. Emergency physicians with less experience took a longer time until they decided to terminate the resuscitation attempt while the specialty of the physician had no influence on the duration of the resuscitations.

## Data Availability

The datasets used and analysed in the current study are available from the corresponding author on reasonable request.
